# Supplementary material for: Prototyping a Knowledge-Based System to Identify Botanical Extracts for Plant Health in Sub-Saharan Africa
Source: Plants (Basel). 2021 Apr 29;10(5):896. doi: 10.3390/plants10050896 (PMC8146496; doi:10.3390/plants10050896)
Supplement: Supplementary file 1 [file plants-10-00896-s001.zip › sup/FigureS1.pdf]

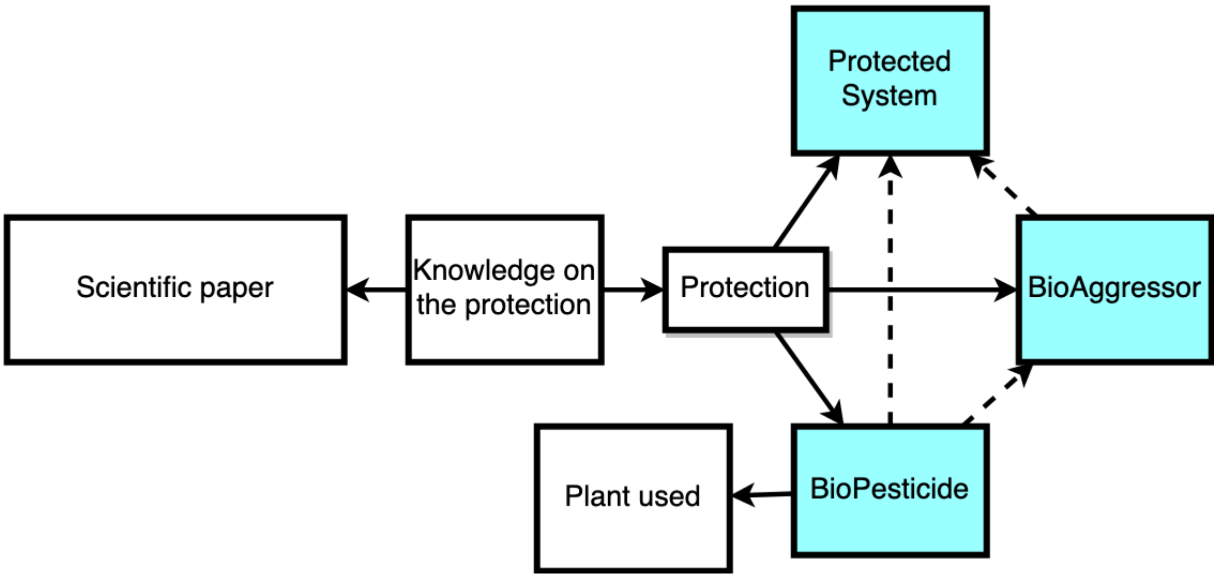

Figure 1. Ontology diagram describing the knowledge base (KB): links between ‘biopesticide’, ‘protected systems’ and ‘organism’ concepts
