# Supplementary material for: Prototyping a Knowledge-Based System to Identify Botanical Extracts for Plant Health in Sub-Saharan Africa
Source: Plants (Basel). 2021 Apr 29;10(5):896. doi: 10.3390/plants10050896 (PMC8146496; doi:10.3390/plants10050896)
Supplement: Supplementary file 1 [file plants-10-00896-s001.zip › sup/FigureS3 Lattice example.pdf]

Figure S3 Lattice example

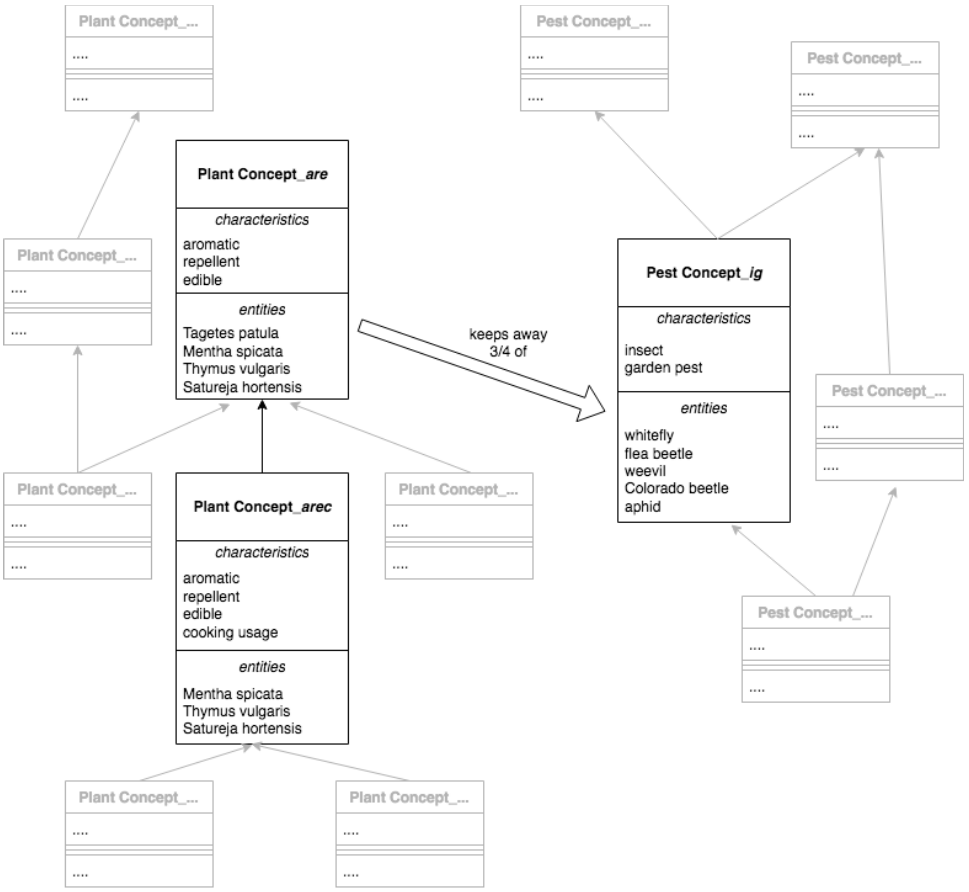

Figure S3 provides a simulated and simplified excerpt of concept lattices on plants (left side) and pests (right side) connected through a cross-lattice relation providing information on repellent effects.

Figure S3 represents a theoretical case to better explain the relationship between groups and the results that can be obtained with the adopted analysis method. According to this theory [152], each group (= rectangular box) is called a concept. In the example, a *plant concept* groups a maximal plant set with the maximal characteristic set they share. For instance, *Plant Concept_are* groups the plant set *Tagetes patula*, *Mentha spicata*, *Thymus vulgaris* and *Satureja hortensis* with the characteristic set aromatic, repellent, edible. In this example, the plants are the classified entities. The subgroup (i.e. a subconcept) *Plant Concept_arec* groups the plant set *Mentha spicata*, *Thymus vulgaris*, *Satureja hortensis* with the characteristic set *aromatic*, *repellent*, *edible* and *cooking usage*. Subgroups contain more characteristics and fewer entities. An arrow from a subgroup (*Plant Concept_arec*) to one of its supergroups (*Plant Concept_are*) means that entities of the subgroup are included in entities of the supergroup and, conversely, supergroup characteristics are included in the subgroup characteristics.

The groups and the specialization relationships between groups support data querying. For example, from a user query identifying *Plant Concept_are* as one group of repellent and edible plants, the concept informs the user that these plants also are aromatic. Taking a look at subgroups highlights that one of them includes the plant commonly used in cooking, thereby providing an opportunity for choosing multipurpose plants.

Figure S3 also draws a pest concept classification, where *Pest Concept_ig* is highlighted. This pest concept groups whitefly, flea beetle, weevil, Colorado beetle and aphid, which are *insects* commonly found in *gardens*, as shown in the characteristic part of the concept.

In this theoretical example, we assume that *Tagetes patula* has been recognized in publications as being able to repel whiteflies, flea beetles, weevils and Colorado beetles, but we do not know if *Tagetes patula* repels aphids. This information is sufficient to establish that *Tagetes patula* repels 3/4 of the pests grouped in *Pest Concept_ig* (hence the arrow is true for *Tagetes patula* as well as for the other plants of *Plant Concept_are*).

In this Figure, *Plant Concept_are* includes plants that share a common relationship to *Pest Concept_ig*: each of these plants is known to be able to repel three quarters of pests grouped in *Pest Concept_ig.* "three quarters of" is an example of a large range of 'relational' operators that can be applied during analysis.

The group *Plant_Concept_are* suggests that all edible aromatic plants it contains repel most of the pests grouped in *Pest Concept_ig*. Thus, this knowledge at the group level can be used (abductive reasoning) at the individual level to hypothesize that *Tagetes patula* repels aphids, and it could therefore be of interest to conduct an experiment to establish whether or not this is the case.
