# Supplementary material for: Prototyping a Knowledge-Based System to Identify Botanical Extracts for Plant Health in Sub-Saharan Africa
Source: Plants (Basel). 2021 Apr 29;10(5):896. doi: 10.3390/plants10050896 (PMC8146496; doi:10.3390/plants10050896)
Supplement: Supplementary file 1 [file FigureS2.pdf › sup/FigureS2.pdf]

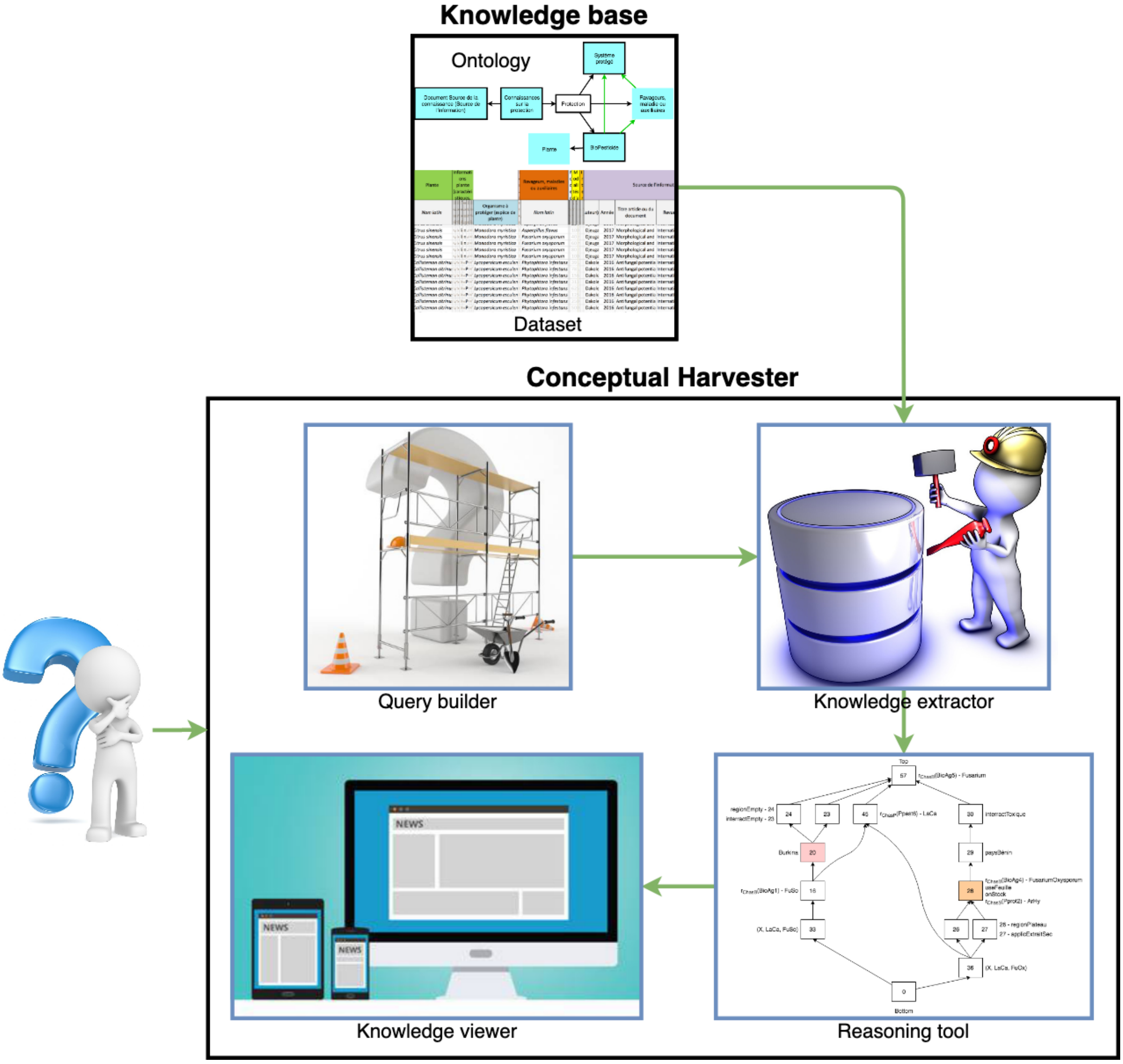

Figure 2. Overall diagram showing relations between the matrix built (dataset), described by an ontology (top part) and the four-element Conceptual Harvester: query builder, knowledge extractor, reasoning tool and knowledge viewer (source [149]).
